# In vivo dose–response analysis to acetylcholine: pharmacodynamic assessment by polarized reflectance spectroscopy

**DOI:** 10.1038/s41598-022-10617-x

**Published:** 2022-04-21

**Authors:** Joakim Henricson, Folke Sjöberg, Fredrik Iredahl, Tomas Strömberg, Daniel Wilhelms

**Affiliations:** 1 Department of Emergency Medicine, Local Health Care Services in Central Östergötland, 58182 Linköping, Sweden; 2 Department of Biomedical and Clinical Sciences, Faculty of Health Sciences, Linköping University, Linköping, Sweden; 3 Department of Hand, Plastic Surgery, Burns and Intensive Care, Linköping University Hospital, 58182 Linköping, Sweden; 4 Department of Health, Medicine and Caring Sciences, Division of Community Medicine, Linköping University, Linköping, Sweden; 5 Department of Primary Health Care, Region Östergötland, 58182 Linköping, Sweden; 6 Department of Medical and Health Sciences, Faculty of Health Sciences, Linköping University, Linköping, Sweden; 7 Department of Biomedical Engineering, Linköping University, Linköping, Sweden

**Keywords:** Drug delivery, Pharmacodynamics, Pharmacokinetics

## Abstract

Transdermal iontophoresis offers an in vivo alternative to the strain-gauge model for measurement of vascular function but is limited due to lack of technical solutions for outcome assessment. The aims of this study were to, after measurement by polarized reflectance spectroscopy (PRS), use pharmacodynamic dose–response analysis on responses to different concentrations of acetylcholine (ACh); and to examine the effect of three consecutively administered iontophoretic current pulses. The vascular responses in 15 healthy volunteers to iontophorised ACh (5 concentrations, range 0.0001% to 1%, three consecutive pulses of 0.02 mA for 10 min each) were recorded using PRS. Data were fitted to a four-parameter logistic dose response model and compared. Vascular responses were quantifiable by PRS. Similar pharmacodynamic dose response curves could be generated irrespectively of the ACh concentration. Linearly increasing maximum vasodilatory responses were registered with increasing concentration of ACh. A limited linear dose effect of the concentration of ACh was seen between pulses. Polarized reflectance spectroscopy is well suited for measuring vascular responses to iontophoretically administrated ACh. The results of this study support further development of iontophoresis as a method to study vascular function and pharmacological responses in vivo.

## Introduction

To assess blood vessel pharmacodynamic responses, the strain-gauge vascular model, which is based on isometric tension measurements in vitro*,* has long been the gold standard^1^. The model is, however, hampered by lack of normal in vivo vascular dynamics such as systemic as well as local neuronal and endocrine effects^2^. Transdermal in vivo microdosing of vasoactive drugs by iontophoresis offers an interesting alternative. Used in conjunction with non-invasive imaging methods, such as laser Doppler flowmetry (LDF), local pharmacodynamic effects in e.g., the skin can be successfully assessed^3–5^. All Doppler based optical measurement techniques are, however, susceptible to movement artifacts and the signal obtained is dependent on both the velocity and the concentration of red blood cells (RBC) within the measurement volume. Further, conventional LDF is linear to the velocity of the RBCs, but not to the concentration, thus, changes in vessel diameter cannot be explicitly analyzed without the use of complex data analysis^6^. Another optical, non-invasive technique for assessing microvascular reactivity is reflectance spectroscopy^7^. Since the signal in polarized reflectance spectroscopy linearly correlates to changes in RBC concentration^8^, it has been proposed that it is more closely related to vessel diameter than the signal generated by laser Doppler. Thus, this better compares to the in vitro strain gauge vascular model.

A recurring discussion concerning iontophoresis has been related to the magnitude of the drug dose (mg) delivered. It is generally considered that the delivered drug dose is directly dependent on electro-repulsion and correlates to the total electrical charge (in milliColoumb, mC) provided during iontophoresis^9,10^. However, other mechanisms of drug transportation have been shown. Especially interesting is that the current during anodal iontophoresis also causes a movement of water known as electro-osmosis^11,12^, that indirectly provides drug transport. The magnitude of the contribution of this co-transport to the net flow of drug into the tissue can be assumed to be proportional to the drug concentration in the electrode chamber. Given the present knowledge of local drug pharmacodynamics^3,7,13,14^ and the concomitant transport of fluid in which the drug is diluted, the drug concentration in the electrode chamber will affect the total vascular response. If the drug concentration is increased in the iontophoretic electrode chamber a linear increase in total vascular response, albeit small, is anticipated to be added by electro-osmosis. Furthermore, the reproducibility of repeated doses (pulses), which is important for the model precision, has not yet been well studied.

There are several aims to this study: (1) To test the usefulness of polarized reflectance spectroscopy for the assessment of vasodilation, in vivo*,* as examined by studying the dose response effect of Acetylcholine (ACh) in repeated iontophoresis; (2) Investigate the effects of five different concentrations of ACh in the electrode chamber, ranging from 1%, most used in vasodilatory studies, to 0.0001%; (3) Examine the pharmacodynamic effect of consecutively administered iontophoretic pulses.

The electrical dose chosen per iontophoretic pulse was 12 mC to reduce the effect of the non-specific vasodilatation caused by use of larger doses^15^. We hypothesized that the sigmoid dose–response (as expressed as ED50) would not be affected by the drug concentration in the electrode chamber, but that increasing drug concentrations would elicit a correlating positive linear increase in the overall vascular response.

## Materials and methods

Sixteen healthy volunteers were screened, and 15 individuals were found eligible for participation according to the study protocol (Inclusion/exclusion see Supplemental material 1). All participants gave their oral and written informed consent to participate and the study protocol was approved by the Swedish Medical Product Agency as well as the Swedish Ethical Review Authority (EudraCT-nbr: 2018-004624-11 and permit number: 2019-01138). The study protocol is published at clinicaltrials.gov (publication date 02/03/2021, NCT04777383). The study described has been carried out in accordance with the code of ethics of the world medical association (declaration of Helsinki) for experiments involving humans.

Acetylcholine (Miochol-E, 10 mg/ml, Baush & Lomb) powder (20 mg) was dissolved to a 1% (0.055 mol/l) solution using 2 ml sterilized water (100 ml sterile water, Braun, Melsungen, Germany). The 1% solution was then diluted further into 0.1% (0.0055 mol/l), 0.01% (0.00055 mol/l), 0.001% (0.000055 mol/l) and 0.0001% (0.0000055 mol/l) solutions using sterilized water. Acetylcholine was chosen since it is a well-established drug to study endothelial-dependent vasodilation and smooth muscle function^16^.

**Figure 1:**
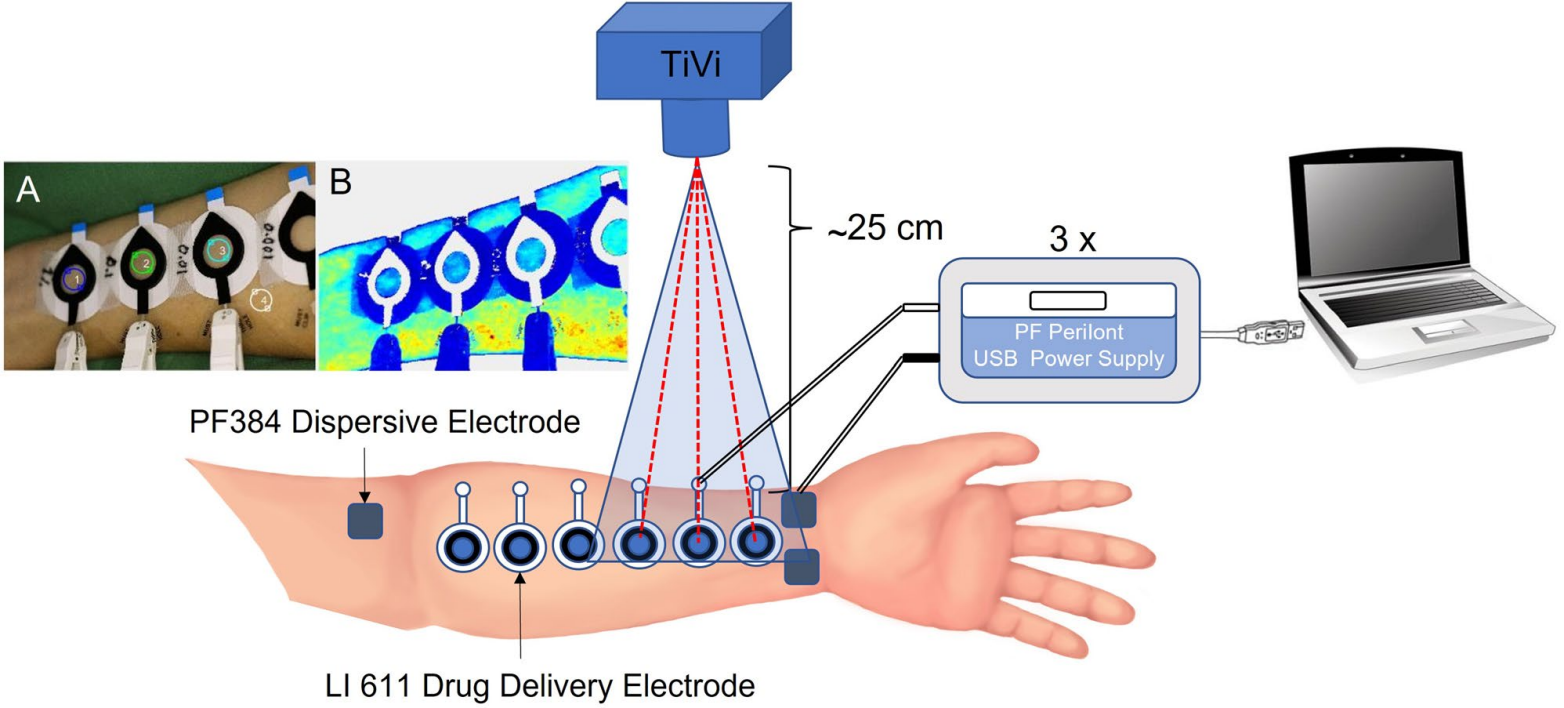
Schematic of the set up for the measurement. Drugs were delivered to three areas simultaneously by three iontophoretic pulses of 10 min each, separated by wash-out periods of 30 min. Local change in red blood cell concentration was measured by polarization spectroscopy (TiVi). (**A**) Shows an example of a regular photo of the measurement areas and the location of the region of interests (blue, green, cyan, and white circles). (**B**) Shows the same measurement areas but in a false color-coded image where red indicates a high TiVi-value and dark blue a low. This figure was made in Microsoft PowerPoint (Microsoft PowerPoint för Microsoft 365 MSO (Version 2202 Build 16.0.14931.20118, Microsoft.com).

Prior the onset of the measurements all volunteers underwent a 20-min acclimatization period to allow their circulation to adapt to the conditions in the test room. Volunteers rested comfortably in a semi supine position with the measurement arm on an arm table at heart level throughout the acclimatization and during the measurements. The volunteers’ skin were gently cleaned using chlorhexidine ethanol (5 mg/mL; Fresenius, Uppsala, Sweden) and let to air dry prior attachment of the drug electrodes. Six drug delivery electrodes (one for each concentration of ACh and one for sterile water) for iontophoresis (LI 611, Perimed AB, Järfälla, Sweden) per subject were attached by double adhesive tape to the volar side of one of the forearms. Three dispersive electrodes, not containing any drug, were also attached to complete the circuit (PF 384, Perimed AB, Järfälla, Sweden) (Fig. 1). The acetylcholine solutions were added to the drug electrode chambers by pipette (~ 350 µl) and care was taken to avoid any air bubbles in the electrode chambers.

**Figure 2:**
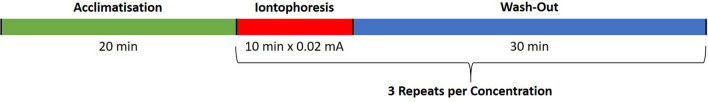
Schematic of the timeline of the experiment. Iontophoresis and wash-out was repeated three times after acclimatization.

Three drug delivery electrodes and three dispersive electrodes were connected to one USB power supply each (LI 760 PeriIont Micropharmacology System, Permied AB, Järfälla, Sweden) (Fig. 1). The acetylcholine solutions were administered using anodal single pulse protocol with a duration of 10 min at a current of 0.02 mA (total electrical charge 12 mC) (Fig. 2). Three different solutions were delivered in parallel by three adjacent drug delivery electrodes. Each drug solution was iontophoretically administered three times with a recovery/wash-out period of 30 min between pulses.

The skin and room temperatures were measured on each occasion before and after the experiment. The conditions in the examination room were kept as constant as possible during the experiments as skin and ambient temperature have an effect on the cutaneous circulation^17^. See Table 1 for more detailed information.

**Table 1:** Demographical data including temperatures.

Subject	Sex	Age (years)	Blood pressure (mmHg)		Pulse (bpm)	Weight (kg)	Height (m)	BMI (kg/m^2^)	Skin temp (°C)		Room temp (°C)	
			Systolic	Diastolic					Before	After	Before	After
1	F	41	104	70	70	50	1.69	18	31.2	28.0	21.4	22.1
2	M	27	119	87	53	88	1.87	25	–*	–*	21.5	21.7
3	F	38	117	83	72	58	1.65	21	30.3	26.7	21.3	21.3
4	M	34	112	72	72	77	1.79	24	31.3	28.9	21.5	22.2
5	M	35	130	86	83	85	1.86	25	31.3	29.3	21.6	22.0
6	F	41	104	81	83	71	1.64	26	30.5	25.5	21.3	22.2
7	F	44	99	67	84	65	1.68	23	30.2	26.7	21.3	21.5
8	F	24	109	76	82	86	1.60	34	30.2	27.6	21.2	21.8
9	F	25	109	78	65	63	1.71	22	32.8	25.9	20.8	20.9
10	F	18	119	81	87	68	1.79	21	30.0	27.7	20.9	21.6
11	M	28	139	84	52	85	1.87	24	30.3	29.4	21.8	22.1
12	M	19	110	72	69	68	1.77	22	31.5	28.4	21.3	21.3
13	F	31	100	70	50	71	1.69	25	30.1	25.5	21.3	22.5
14	F	26	112	70	70	62	1.75	20	30.5	28.5	21.5	22.5
15	M	27	123	78	69	94	1.90	26	31.3	28.2	20.5	21.7
**Means (± SD)**												
All	15	30.5 (8.0)	114 (11)	77 (7)	71 (12)	72.7 (12.6)	1.8 (0.1)	24.0 (3.7)	30.8 (0.8)	27.6 (1.3)	21.3 (0.3)	21.8 (0.5)
Males	6	28.3 (5.8)	122 (11)	80 (7)	66 (12)	82.8 (9.1)	1.8 (0.1)	24.0 (1.5)	31.1 (0.5)	28.8 (0.5)		
Females	9	32.0 (9.3)	108 (7)	75 (6)	74 (12)	66.0 (10.0)	1.7 (0.1)	23.3 (4.6)	30.6 (0.9)	26.9 (1.1)		

*Missing data due to broken thermometer.

Vascular responses were quantified using polarized reflectance spectroscopy by tissue viability imaging (TiVi700 2.0 Tissue Viability Imager, WheelsBridge AB, Linköping, Sweden). The technique has previously been described in detail^8,18^. In short, the system comprises a high-performance digital camera equipped with perpendicular oriented polarization filters in front of the light source and the camera lens. The polarization eliminates specularly reflected light and enables imaging of the dermal layer for information about the concentration of RBCs in the microvasculature. The depth at which the incident linearly polarized light has become almost completely randomly polarized is typically in the order of 300 µm, i.e., well into the reticular dermis in most skin sites. The approximated maximum measurement depth is 1.8 mm (for light in the red wavelength region)^19^. The RBCs in the microcirculatory network of the skin strongly absorb light in the green (approximately 500–600 nm) wavelength region, while light in the red (approximately 600–700 nm) wavelength region is less absorbed. Since the light absorption of the static tissue components in dermis is smaller and less dependent on wavelength than the light absorption in RBCs, visible light spectroscopy can be used for the in-vivo evaluation of RBC content and the associated tissue viability.

The technology takes advantage of the wavelength dependence in RBC absorption. By first separating the red, green, and blue color matrixes and then applying an algorithm in which the value of each picture element in the green color matrix is subtracted from corresponding value of the red color matrix, an output matrix linearly proportional to the local RBC concentration can be generated^8,18^. 

Image analysis and calculation of TiVi-values were made using WheelsBridge AB Software, version 1.2.20, November 2018, Linköping, Sweden. The tissue viability imager was set to single photo mode and medium resolution and positioned at approximately 25 cm above the active drug delivery electrodes. This setup allowed for three adjacent drug electrodes to be photographed simultaneous. An image of the three adjacent electrodes was taken prior the onset of iontophoresis (one baseline image per pulse) and then every 60 s for the duration of the drug delivery (3 × 10 min) and wash-out periods (3 × 30 min) resulting in 123 images per three electrodes, and 246 images per subject in total.

### Data analysis and statistics

Four regions of interest, three covering the inner circle of each of the active drug electrodes (~ 1.1 cm^2^ each) per iontophoretic pulse and one in a control site (unaffected skin) near the test sites, were applied to all photographs, generating values correlating to the local RBC concentrations within those regions. Responses were adjusted to show only relative change by subtracting the value of the first (baseline) image in each data set from the subsequent values. Non-responders were defined as areas with a maximum relative increase of less than 10 units. Such measurements were excluded from further analysis, see Supplementary material 2 for more details. Remaining data was pooled and normalized between zero and hundred percent by creating means at every timepoint. In this new column of pooled mean values, “zero” was defined as the first value and “hundred” as the highest value. Graphs for mean relative change in absolute numbers and for the normalized mean data (Fig. 4) for each pulse and concentration of drug were plotted over time as dose response graphs (Fig. 5). One-way repeated measures ANOVA was used to test between baseline values and response after 10 min of iontophoresis for respective pulse. Only data from the baseline image and the ten following images captured during the iontophoresis were used for dose response analysis. An agonist vs. response, four-parameter logistic curve model (min and max restricted to 0 and 100, respectively) was fitted to the data to generate best fit values for Hill slope and ED50 (Effective Dose 50%, pharmacodynamically assessed as time (min) to 50% of maximum response). ED50 values for the dose response curves for the pulses and concentrations were compared using an extra-sums-of-squares *F* test to find any difference between data sets.

Dose response curves and statistical analysis were plotted and performed in GraphPad Prism version 8.4.3 for Windows, GraphPad Software, San Diego, California USA, http://www.graphpad.com.

A p-value of ≤ 0.05 was considered as significant in all calculations.

## Results

Demographic data of the healthy volunteers and room temperature are presented in Table 1.

No adverse events were observed or reported by the participants. The iontophoretic administration of ACh elicited a relative increase compared to baseline values larger than 10 units in 165 (73%) of the 225 measurements. Leakage from the iontophoresis chamber, as confirmed by the photographs taken by the TiVi system, was the cause to non-responsiveness in seven of the 60 drug delivery sites. No obvious reason could be found for the lack of response for the remaining 53 measurements (Supplemental material 2).

### Baseline analysis

ANOVA repeated measures analysis showed that the pulse number significantly affected the TiVi value (p < 0.001), where pairwise comparisons showed that TiVi values for pulses 2 and 3 were significantly higher than that for pulse 1 (p < 0.001). Furthermore, dose significantly affected TiVi values (p < 0.05). However, there was an interaction effect between pulse number and dose, where values for pulse 1 and pulse 2 showed no effect of dose, but for pulse 3 baseline increased with decreasing dose. These data show that the washout period was insufficient for TiVi values to return to the values before the iontophoresis.

### 10-min responses

ANOVA repeated measures showed that concentration of ACh in the drug chamber significantly affected TiVi-values for each of the pulses (p < 0.05), where a higher concentration consequently yielded a higher TiVi value. For pulse 1, TiVi-values for concentration 0.0001% (p < 0.05) and 0.001% (borderline, p = 0.06), were smaller than for concentration 1%. For pulse 2, TiVi-values for concentration 0.0001% (p < 0.05) and 0.00 1% (p < 0.05) and 0.1% (p = 0.06), were smaller than for concentration 1%. For pulse 3, TiVi-values for concentration 0.0001% (p < 0.001), 0.001% (p < 0.001) and 0.01% (p < 0.01), were smaller than for concentration 1%.

**Figure 3:**
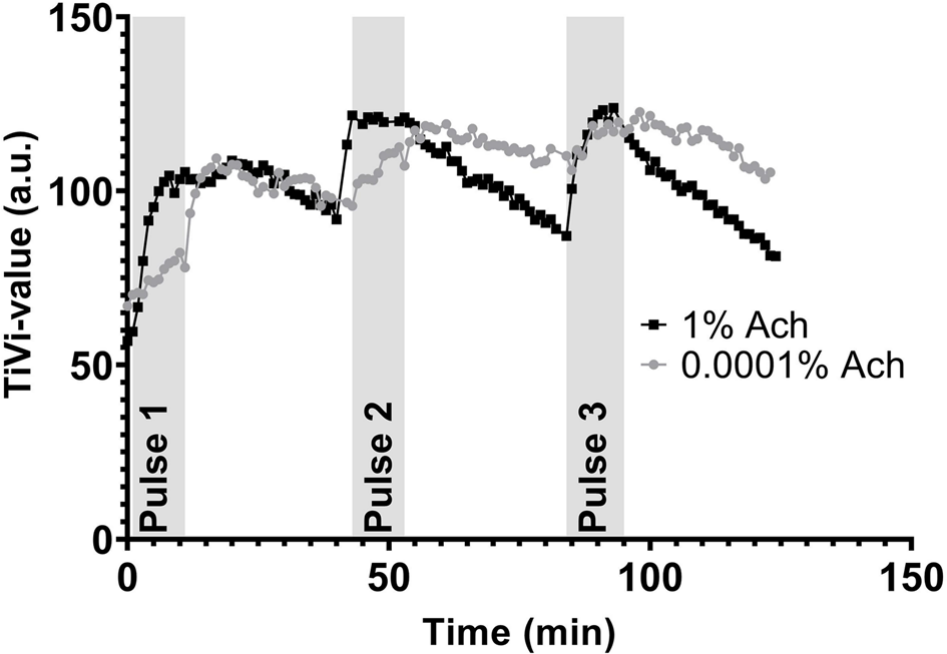
A typical example from one subject of changes in red blood cell concentration, as measured by polarization spectroscopy, during repeated pulses (Pulses 1 to 3) of iontophoresis and periods of following wash-out of the lowest and highest drug concentrations (0.0001% and 1% ACh).

A typical response from one subject during the whole observation period (baseline to wash-out pulse 3) for ACh in the concentration of 1% and 0.0001% is seen in Fig. 3. For the highest concentration of ACh (1%) there was a significant increase from baseline to the end of the iontophoretic pulse 1 (p < 0.0001), and as well between baseline for before pulse 1 and baseline before pulse 2 (p = 0.037), indicating an insufficient recovery period. No significant difference was seen between baseline before pulse 1 compared to the baseline before pulse 3 (p = 0.92), indicating a more rapid recovery period after the second pulse. Both the second and third iontophoretic pulse led to a significant increase compared to the respective baseline before the start of the respective pulses (pulse 2: p = 0.0034 and pulse 3: p < 0.0001).

For the lowest concentration of ACh (0.0001%) there was a significant increase from baseline to the end of the iontophoretic pulse 1 (p < 0.0001), as well as baseline before pulse 2 and pulse 3 (pulse 2 p = 0.0002, pulse 3 p < 0.0001), indicating an insufficient recovery period. Both the second and third iontophoretic pulse led to a significant increase compared to the respective baseline before the start of the pulse (Pulse 2: p = 0.0002 and Pulse 3: p = 0.0034).

**Figure 4:**
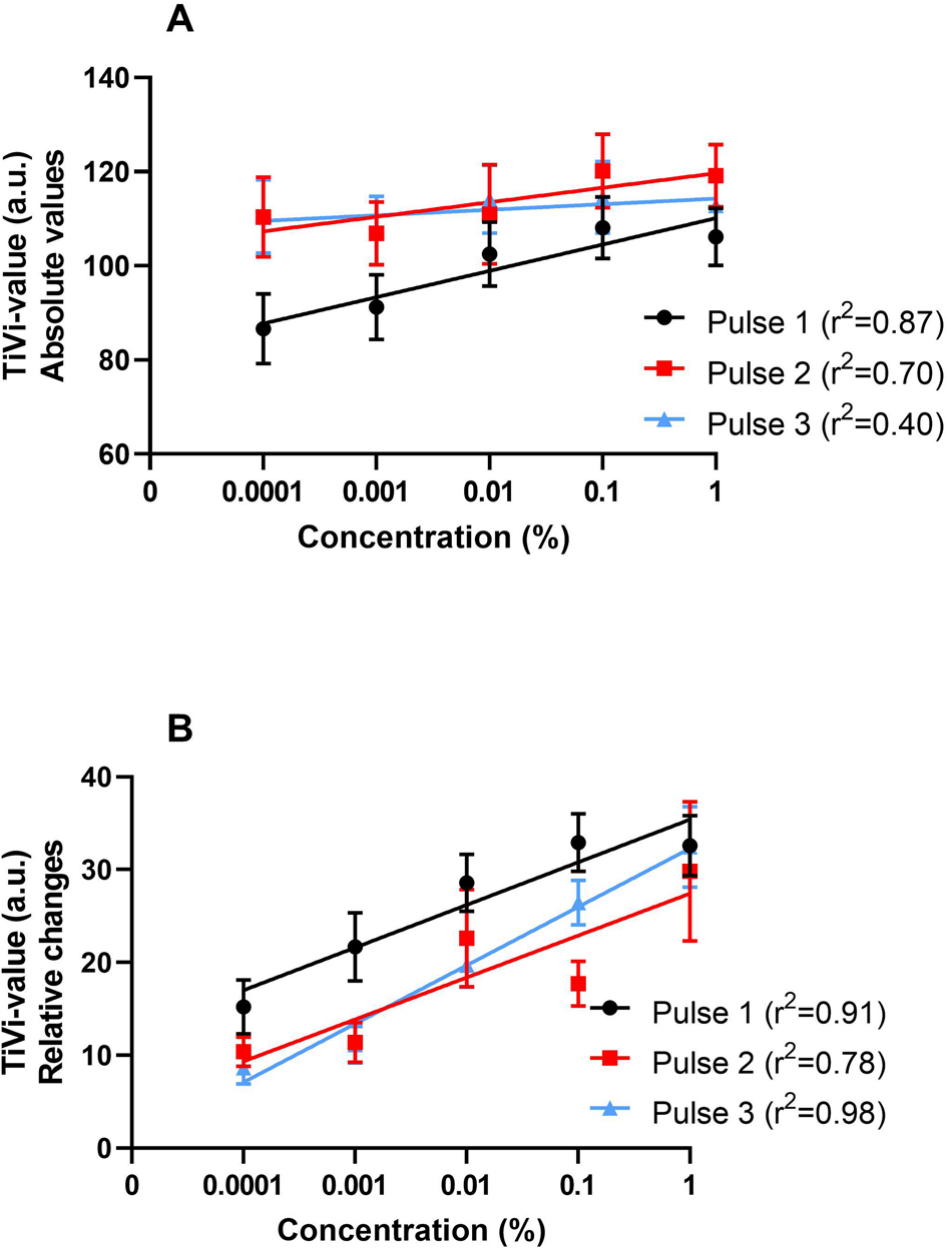
Absolute mean values (**A**) and baseline reduced mean values (**B**) (n = 15) of changes in red blood cell concentration as measured by polarization spectroscopy after iontophoretic delivery of acetylcholine of five concentrations. Acetylcholine was delivered during 10 min at three repetitions separated by 30 min wash-out between each iontophoretic pulse (1–3). Relative changes (**B**) as compared to baseline before the start of each pulse. Error bars show standard error of the mean (SEM).

**Figure 5:**
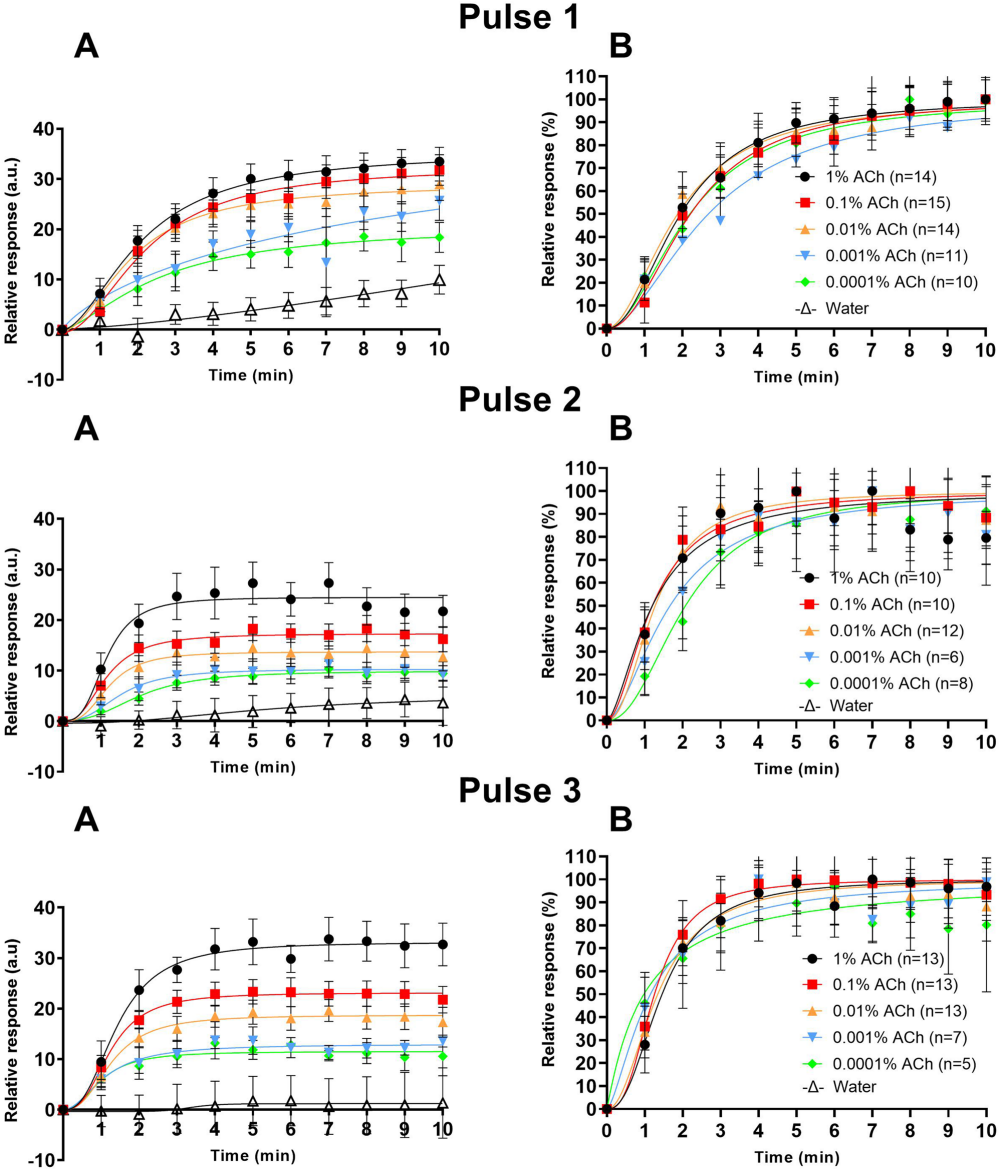
Baseline reduced mean values (**A**) and baseline reduce and normalized (**B**) dose–response curves of red blood cell concentration as measured by polarized spectroscopy (TiVi), before and during iontophoresis of acetylcholine in five concentrations and control solution (sterilized water). n = 15. Acetylcholine was delivered during 10 min at three repetitions separated by 30 min wash-out between each iontophoretic pulse (1–3). Error bars show standard error of the mean (SEM).

A linearly increasing red blood cell concentration after iontophoresis was observed with increasing concentration (range 0.0001–1%) of ACh in the drug chamber (Fig. 4A,B) for all pulses. The linear regression based on absolute values (Fig. 4A) resulted in R^2^-values of 0.87, 0.70 and 0.40 for Pulse 1, 2 and 3, respectively. The R^2^-values for the change in relative values (Fig. 4B) were 0.91, 0.78, 0.98 for Pulse 1, 2 and 3, respectively. Similar pharmacodynamic dose response curves (ED50 values) could be generated irrespectively of the ACh concentration (range 0.0001–1%) in the drug chamber (Fig. 5). Best fit values for ED50 for the pooled curves of each pulse and concentration are presented in Table 2. The extra-sums-of-squares *F* test for best fit values for Hill slope and ED50 values between pooled dose response curves of different concentrations for each pulse showed no significant difference (p values 0.57 for Pulse 1, 0.63 for Pulse 2, and 0.81 for Pulse 3).

**Table 2:** Best fit values for ED50 (95% CI within parentheses) from the agonist vs. normalized response variable least square fit analysis performed on pooled red blood cell concentration data from transdermal iontophoresis of acetylcholine as measured by polarization spectroscopy. ED50 expressed as time (min) to 50% of the full (100%) response after each iontophoretic pulse (1–3) during iontophoresis (10 min).

ED50	1% ACh	0.1% ACh	0.01% ACh	0.001% ACh	0.0001% ACh
Pulse 1	2.0 (1.5–2.4)	2.2 (1.7–2.7)	1.9 (1.4–2.4)	2.8 (1.5–4.0)	2.2 (1.3–3.1)
Pulse 2	1.2 (0.29–1.8)	1.2 (0.59–1.7)	1.3 (0.41–1.9)	1.7 (0.48–2.6)	2.1 (1.4–2.7)
Pulse 3	1.5 (1.0–1.9)	1.3 (0.93–1.6)	1.3 (0.85–1.8)	1.1 (0.19–1.9)	0.98 (0.019–1.8)

## Discussion

This study presents new and interesting data that supports further development of iontophoresis as a method to study vascular function in vivo, in humans.

Firstly, the data suggests that the new application, by which polarized reflectance spectroscopy rather than laser Doppler methodology, provides relevant iontophoretic dose–response effects, as assessed as ED50 values of iontophoretically applied ACh in skin is working well. We chose to assess the drug response with TiVi based on its ability to identify RBC independent of movement (vascular dimension). The more established method LDF has a relatively high site-to-site variability^13^ possibly affecting the interpretation of results. TiVi is also relatively inexpensive, potentially facilitating a more widespread use. Given the relative simplicity of both the model and its readouts, we believe that the use of polarized reflectance spectroscopy can facilitate an earlier transition to in vivo studies of the vascular properties of future drug candidates.

Acetylcholine delivered by the transdermal iontophoresis current elicits a dose-dependent (current × time) vascular dimension increase, seen by the TiVi as an increase in red blood cell concentration. The findings in the present study mimics closely the results obtained previously with LDF^20^. No significant differences in ED50 were seen between the same current dose (dose–response) given as repeated pulses.

An important observation in this investigation using TiVi rather than traditional LDF is that the maximum dilatory response at all chambers containing ACh concentrations (1–0.0001%) was found after a total charge (current × time) of 6 mC. This contrasts the findings using conventional LDF by which a larger dose (charge) of 12 mC is needed to reach the response plateau^20^. The advantage in the present setting being that with increasing doses there is risk for a concomitant non-specific vasodilatation caused by the current/vehicle and which is known to negatively affect the interpretation of the results^15,21^. The discrepancy between these two findings is difficult to ascertain, but it may be speculated that in the case of the TiVi the method mainly depicts the early changes in diameter of the vessels whereas LDF is dependent of both the number of RBCs and their velocity. Since the velocity of the RBCs is reduced as the diameter of the vessels increase and the number of RBCs increase during vasodilation, the LDF may not detect initial changes of vessel diameter. Also, when examining the effect of a strict water iontophoresis as control to ACh the water effect was found negligible as compared with LDF measurements^15^. Therefore, TiVi offers comparable results to the in vitro vascular strain-gauge model and may be claimed preferable in local pharmacodynamic assessment of vascular diameter response.

Secondly, an increasing vasodilatory effect was registered in consecutive pulses after the first current pulse. The decreased dose needed in the second and third delivery cycles to reach ED50 highlights the skin’s natural adaption process and is in line with our previous study^20^, but could also be partly explained by insufficient recovery to pre iontophoresis baseline conditions. These results emphasize the importance of establishing in vivo models where changes in local environment can be followed in real time to understand the mechanism leading to vascular regulation to different provocations.

Acetylcholine is a muscarinic receptor agonist which is degraded by hydrolysis by acetylcholinesterase mainly located in the blood compartment. Possible explanations to the increased response could be decreased electrical resistance of the skin facilitating drug transport^22^, and/or altered drug degradation and/or affected receptor sensitivity. Since perturbations in vascular control of the smaller vessels in the skin have been suggested to occur before systemic effects can be observed^23^, it is likely that local pharmacological reactions to drugs is a sensitive mirror of possible also systemic vascular effects.

Thirdly**,** linearly increasing maximum total iontophoretic responses were registered with increasing concentration (range 0.0001–1%) of ACh in the drug chamber (Fig. 4A). Given that the electro-osmotic fluid volume delivered to the tissue is similar between each experiment (the total charge being the same, 12 mC, in all experiments); the higher concentration of the drug in the fluid transported by electro-osmosis, i.e., in the 1% solution will lead to a larger total deposit of ACh in the tissue, together with a fluid volume affecting a larger tissue volume than that of electro-repulsion/electromigration, and thereby dilating more vessels and concomitantly generating at larger total TiVi value. The linear relationship between maximum vascular response and drug chamber concentration supports this reasoning.

Fourthly, contrarily to the electro-osmotic effect, similar pharmacodynamic dose response curves (ED50) were generated irrespectively of the ACh concentration in the drug chamber. These response effects are strictly current related (electro-repulsion/electromigration) and the local concentration of the drug in the tissue is directly proportional to the current. The sigmoid curve depicts the pharmacodynamic properties of the ACh receptor interaction and its vascular response^3^.

Fifthly, a small linear dose effect, absolute TiVi values 20 (a.u) (Fig. 5A) of the concentration of ACh in the drug chamber was seen that remained before the start of the second and third pulse. The origin of this finding is unclear but may reflect remaining ACh in the larger extravascular compartment not accessible to acetylcholinesterase and slowly diffusing into the vascular compartment and causing vasodilation between pulses.

## Limitations

### The iontophoretic model

In the present model there were 60 of 225 provocations that did not cause a response. This is a regular finding in most iontophoresis experiments and the number in this study was less when subtracting situations where a leak from the electrode chamber was the explanation. However, non-responders were still present. This issue has been discussed before and no convincing overall conclusion has yet been presented. One factor that is thought to be the most important is that in the present vasodilatory experiment, the blood vessels are not in a pre-constricted state, but vasoconstriction/dilatation vary locally and over time. This is different to e.g., the in vitro strain-gauge model where a vasoconstriction is made by potassium or noradrenaline prior to the vasodilatation experiment. Another issue based on the same reasoning is for the repeated pulses, as the vasodilation from a previous pulse may still to some extent be present despite a 30-min washout period. This reasoning is supported by a slight increase in the non-responders seen in the latter, especially last pulse in the study. Compared to the strain-gauge model^1^, drugs delivered by transdermal iontophoresis in combination with live measurements of skin vascular response, offers a standardized and reproducible technique to test vasoactive drugs in live subjects. One strength with live tissue observations is the maintained systemic blood circulation, which enables a wash-out effect based on increased blood flow and local hormonal as well as neurogenic effects. Thus, the effects of transdermal drug administration are similar to systemic drug administration than can be achieved in an in vitro strain-gauge model.

### The iontophoretic technique

This study is also limited, as previous iontophoretic in vivo human studies, by the inability to determine the exact drug dose delivered to the tissue. The drug is in theory thought to be delivered to the tissue by either electro-repulsion/electromigration, electro-osmosis and/or by so called damage effect (current induced increase in skin permeation). We can so far only speculate regarding to the respective contribution of these mechanisms to the total delivered drug dose.

Another limitation is that the results presented only encompasses one drug with advantageous vascular properties for iontophoresis. Further research is needed to expand the knowledge encompassing other drugs or group of drugs with such effects. The present study, albeit not fully answering all these crucial issues, still for the first time try to describe and differentiate some of their possible roles in an in vivo human model.

## Conclusions

Polarized reflectance spectroscopy properly depicts iontophoretic dose–response effects, assessed as ED50 values, of iontophoretically applied ACh in skin. Linearly increasing maximum iontophoretic responses were registered with increasing concentration (range 0.0001–1%) of ACh in the drug chamber. The findings further support transdermal iontophoresis as a useful method to depict vascular drug effects in vivo in humans.

## Supplementary Information

Supplementary Information 1.

Supplementary Information 2.

## Data Availability

The generated and analyzed datasets for this study can be found in the clinicaltrials.gov https://clinicaltrials.gov/ct2/show/NCT04777383?cond=iontophoresis&draw=3&rank=35. ClinicalTrials.gov Identifier: NCT04777383.
