# Supplementary material for: In vivo dose–response analysis to acetylcholine: pharmacodynamic assessment by polarized reflectance spectroscopy
Source: Sci Rep. 2022 Apr 21;12:6594. doi: 10.1038/s41598-022-10617-x (PMC9023454; doi:10.1038/s41598-022-10617-x)
Supplement: Supplementary file 1 — Supplementary Information 1. [file 41598_2022_10617_MOESM1_ESM.docx]

**Supplement 1.**

**Inclusion Criteria:**

- Age between 18 and 45 years
- Generally healthy and without ongoing medication (contraceptives excluded)
- No known or ongoing skin disease or other skin disorders
- Voluntary participation

**Exclusion Criteria:**

- The research person is incapable to understand the oral and written information
- The research person does not sign the informed consent
- Ongoing medication of any kind (contraceptives excluded)
- Known hypertonia, skin disease, cardiovascular disease or pregnancy
- Damaged, bruised, scarred or tattooed skin on both forearms
- Smoker (smoked within 6 months or more than 100 cigarettes)
- User of snus (used snus within 6 months)
- Used any kind of nicotine product (gum, patch or similar) 6 months prior the onset of the test
- Blood pressure above 140/90
- Not refrained from coffee, tea, alcohol, or strenuous physical exercise on the day of the test
- Not fasting 2 hours before the onset of the test
